# An unusual case of total ophthalmoplegia

**DOI:** 10.4103/0301-4738.55072

**Published:** 2009

**Authors:** Ravindra Kumar Chowdhury, Navnit Gupta, Krishna Charan Padhy

**Affiliations:** J.P.M. Rotary Eye Hospital & Research Institute, C.D.A. Sector-6, Bidanasi, Cuttack - 753014, India

**Keywords:** Intracranial foreign body, superior orbital fissure syndrome, traumatic ophthalmoplegia.

## Abstract

An eight-year-old male child presented with drooping of the left eyelid with a history of penetrating injury of hard palate by an iron spoon seven days ago, which had already been removed by the neurosurgeon as the computed tomography scan revealed a spoon in the left posterior ethmoid and sphenoid bone penetrating into the middle cranial fossa. On examination, visual acuity was 20/20 in each eye and left eye showed total ophthalmoplegia. Oral cavity revealed a hole in the left lateral part of the hard palate. We managed the case with tapering dose of systemic prednisolone. The total ophthalmoplegia was markedly improved in one month. Cases of foreign bodies in the orbit with intracranial extension are not unusual, but the path this foreign body traveled through the hard palate without affecting the optic nerve, internal carotid artery or cavernous sinus makes an interesting variation.

Cases of unexpected foreign body in the brain penetrating through the orbit presenting as total ophthalmoplegia have been reported in the literature.[1–3] However, a foreign body penetrating through the hard palate into the brain presenting as total ophthalmoplegia has never been reported as per our knowledge and thorough Medline search. The material of the penetrating foreign body was wood in the previous reported cases whereas in our case, we found it to be an iron spoon.

## Case Report

**Figure 1:**
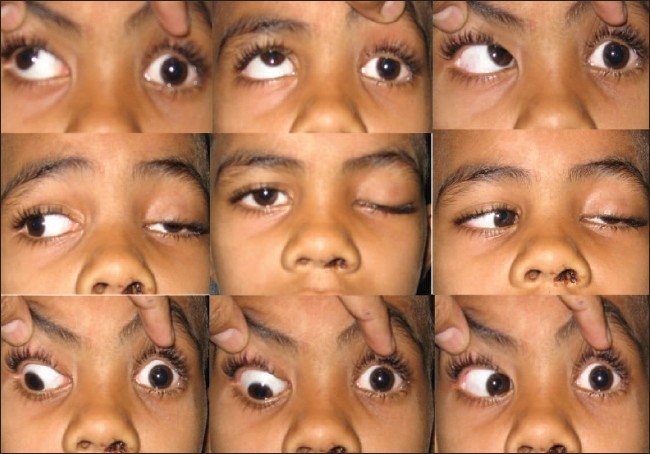
Pretreatment photograph of left total ophthalmoplegia

**Figure 2:**
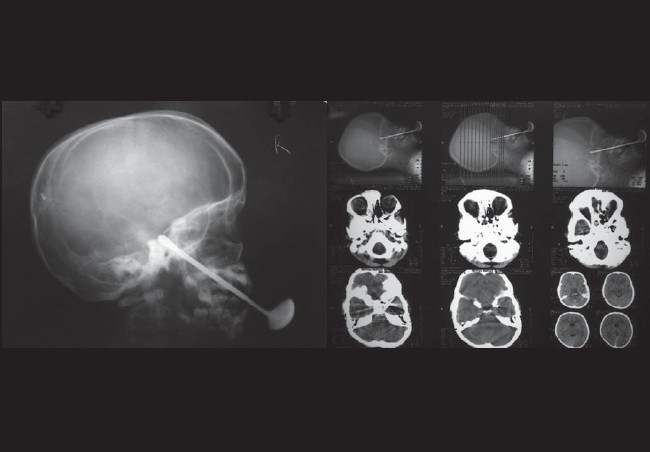
X-ray skull and CT scan of brain and orbit showing entry of spoon through hard palate with intracranial extension

An eight-year-old male child attended the outpatient department, complaining of drooping of the left eyelid [Fig. 1] with a history of penetrating injury of the hard palate by an iron spoon seven days back. The injury had occurred while the child had fallen down with the spoon in his mouth. The iron spoon had already been removed by the neurosurgeon under general anesthesia elsewhere before the child presented to us. The X-ray and computed tomography (CT) scan revealed a radio-opaque spoon perforating the hard palate, passing through the left posterior ethmoid and sphenoid bone reaching into left middle cranial fossa [Fig. 2]. The patient did not complain of any fever, chill or rigor. There was no history of seizures or loss of consciousness. However, bleeding from the nose was reported by the patient. The drooping of eyelid was present before removal of the iron spoon as told by the child's father. This was confirmed by the discharge report of the neurosurgeon, mentioning the presence of ptosis of the left eye and total ophthalmoplegia before removal of the foreign body.

On local examination, the left eye showed complete ptosis with levator function of 2 mm [Fig. 1]. Orbital margins were normal. Extra-ocular movements were absent in all directions of gaze [Fig. 1]. The lid and conjunctiva were normal. Corneal sensation was impaired. The pupil was dilated and fixed. Both direct and consensual light reflex were absent in the left eye and were intact in the right eye. Other anterior segment and posterior segment details were within normal limits. The visual acuity in each eye was 20/20. Color vision of both eyes was intact. The cranial nerve examination of left eye showed third, fourth and sixth nerve palsy along with the involvement of the ophthalmic division of trigeminal nerve.

**Figure 3:**
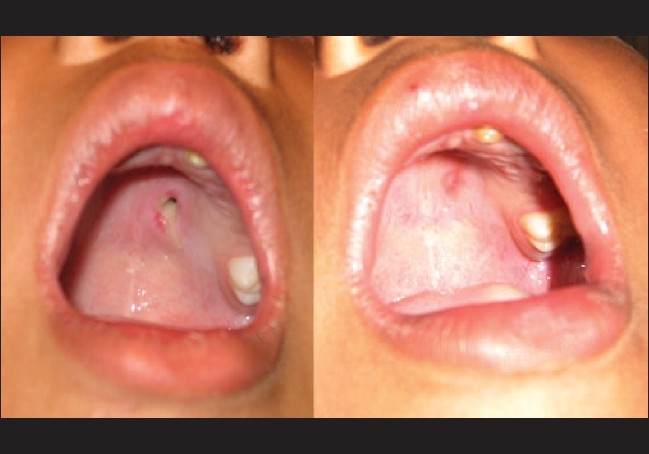
Pretreatment and post-treatment photograph of hole in palate

On examination of the oral cavity, a small full thickness hole of size 1.5 mm×1 mm in the left lateral part of hard palate was seen [Fig. 3]. Hemoglobin, differential count, total leucocyte count and fasting blood sugar were done which were within normal limits.

**Figure 4:**
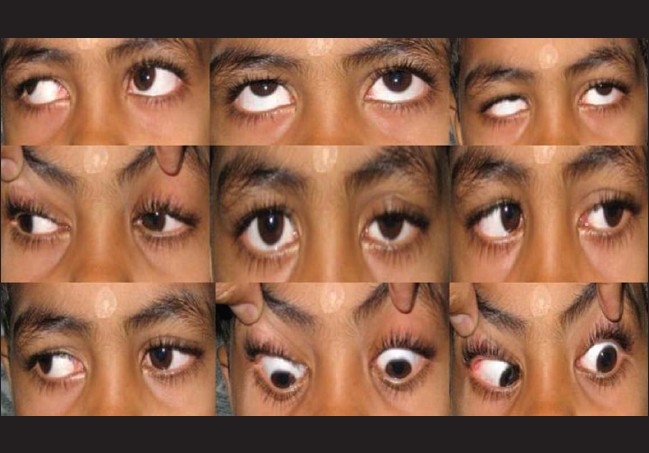
Marked improvement of total ophthalmoplegia following treatment

As the patient came to us with total ophthalmoplegia, we managed the case as left superior orbital fissure syndrome following trauma and started systemic prednisolone empirically with a dose of 1 mg/kg body weight and tapered over a period of one month. Marked improvement of symptoms with closure of the hole in the hard palate was seen in one month follow-up [Fig. 4].

## Discussion

This case presents the clinical course of a child who sustained injury with an iron spoon. Cases of retained foreign bodies in the orbit with intracranial extension are not so unusual,[1–3] but the path which this foreign body traveled makes an interesting one. After having pierced the hard palate, it passed through the posterior ethmoid and sphenoid bone to enter the cranium, narrowly missing the carotid artery and optic nerve. The development of ptosis, ophthalmoplegia and impaired corneal sensation could be explained as direct injury to structures exiting through the superior orbital fissure. The superior orbital fissure syndrome is a rare complication of craniomaxillo facial trauma. The neurological symptoms are generally due to reversible neuropathy caused by edema, contusion and compression of nerves.[4] Improvement of neurological symptoms following post-traumatic superior orbital fissure syndrome have been reported following mega dose of corticosteroid.[5,6] Some authors advocate emergency optic nerve decompression as another treatment modality. However, the study of Sabri *et al.* finds satisfactory results and no complications attributable to mega doses of corticosteroids.[5] Marked improvement of ophthalmoplegia following steroid administration in our case justifies the diagnosis, as the improvement is due to resolution of edema and contusion caused by the trauma. Here a question arises about the cause of the injury, whether directly due to the foreign body or during the surgery for the removal. However, the history and the data in the discharge report clarified that the impairment was present before the surgery. The absence of cavernous sinus thrombosis, optic nerve injury or injury to internal carotid artery, despite the close proximity of this foreign body highlights this rare case.
